# Identification of average molecular weight (AMW) as a useful chemical descriptor to discriminate liver injury-inducing drugs

**DOI:** 10.1371/journal.pone.0253855

**Published:** 2021-06-25

**Authors:** Yuki Shimizu, Takamitsu Sasaki, Jun-ichi Takeshita, Michiko Watanabe, Ryota Shizu, Takuomi Hosaka, Kouichi Yoshinari

**Affiliations:** 1 Laboratory of Molecular Toxicology, School of Pharmaceutical Sciences, University of Shizuoka, Shizuoka, Japan; 2 Research Institute of Science for Safety and Sustainability, National Institute of Advanced Industrial Science and Technology (AIST), Tsukuba, Japan; Universidad de Valladolid, SPAIN

## Abstract

Drug-induced liver injury (DILI) is one of major causes of discontinuing drug development and withdrawing drugs from the market. In this study, we investigated chemical properties associated with DILI using in silico methods, to identify a physicochemical property useful for DILI screening at the early stages of drug development. Total of 652 drugs, including 432 DILI-positive drugs (DILI drugs) and 220 DILI-negative drugs (no-DILI drugs) were selected from Liver Toxicity Knowledge Base of US Food and Drug Administration. Decision tree models were constructed using 2,473 descriptors as explanatory variables. In the final model, the descriptor AMW, representing average molecular weight, was found to be at the first node and showed the highest importance value. With AMW alone, 276 DILI drugs (64%) and 156 no-DILI drugs (71%) were correctly classified. Discrimination with AMW was then performed using therapeutic category information. The performance of discrimination depended on the category and significantly high performance (>0.8 balanced accuracy) was obtained in some categories. Taken together, the present results suggest AMW as a novel descriptor useful for detecting drugs with DILI risk. The information presented may be valuable for the safety assessment of drug candidates at the early stage of drug development.

## Introduction

Hepatotoxicity is one of major causes of drug withdrawal from the market [1–3]. It is very difficult to detect liver injury potential in the early stage of drug development since the incidence of drug-induced liver injury (DILI) is very low, that is 1 in 50,000 to 1 in 100,000 patients (0.001% - 0.002%) [4]. In addition, molecular mechanisms of DILI are barely understood and thus no valid predictive evaluation system has been established for DILI.

Various factors are associated with the onset of DILI. One of the major causes of DILI is the formation of reactive metabolites, which bind to and denature macromolecules, such as proteins and lipids, to induce cellular dysfunction, inflammatory stress and allergic reactions, and thereby toxicological responses [4–6]. It is also known that there are large inter-individual variations in the clinical symptom of DILI, which mainly result from the patient-related factors [7, 8]. For example, genetic polymorphisms of genes associated with drug metabolism and immune/inflammatory response have been identified as risk factors for DILI [9], and the involvement of the haplotypes of human leukocyte antigen in DILI has been reported [10].

A variety of in vitro assays have been established to assess the toxicological potency of drugs at the cell level, such as those using a high-content screening (HCS) method [11] or monocytes to detect immune/inflammatory responses [12]. In addition, high-lipophilicity (e.g. logP ≥ 3) [13] and high daily dose (e.g. >50–100 mg per day) [14] as well as the above-mentioned reactive metabolite formation by drug-metabolizing enzymes, such as cytochrome P450s and UDP-glucuronosyltransferases [15], have been suggested as DILI-inducible factors [16, 17]. Therefore, a DILI prediction by the combination of HCS and the rule-of-two (i.e. daily dose ≥ 100 mg/day and logP ≥ 3) was tested and it showed improved prediction accuracy and reduced the number of drugs that were subjected to further experimental assessments, compared to a prediction by HCS alone [18]. These combination approaches are practical and employed in many pharmaceutical companies for drug development. However, they may not be applied to the early drug discovery stage since it requires daily dose information and synthesis of candidate compounds.

In silico methods have been applied to the toxicity prediction of chemical substances such as pharmaceuticals [19]. Toxicity predictions based on in silico methods can be performed at lower costs and in high-throughput systems, and they do not require chemical synthesis and in vivo and in vitro experiments. In silico approaches are thus ideal for screening at the early stage of drug development. In these studies, parameters (i.e. chemical descriptors) calculated from physiochemical properties of drugs are used, which can be easily obtained as quantitative data from chemical structures [20, 21].

Decision tree-based analysis is a classical classification method of machine learning [22]. While its predictive performance is usually lower than that of other related methods, such as deep learning and random forest, it has an advantage that a constructed model has readability. Moreover, the resulting information on partial chemical structures and physicochemical properties that contribute to toxicity will be valuable for the feedback to medicinal chemistry to reduce toxicity through synthetic development at the early stage of drug development.

The aim of this study was to identify a physicochemical property that is simple to understand and useful for DILI screening in the early stage of drug development. To this end, we investigated chemical properties associated with DILI, using decision tree-based methods with DILI-positive drugs (DILI drugs) and DILI-negative drugs (no-DILI drugs) selected from Liver Toxicity Knowledge Base (LTKB) [23, 24]. Chemical descriptors associated with partial chemical structures, lipophilicity, surface charges and others were calculated as indices of physicochemical properties of the drugs and we then sought to identify an important factor(s) to discriminate DILI drugs from no-DILI drugs.

## Materials and methods

### Dataset

The DILIrank dataset, which consists of 1,036 US Food and Drug Administration (FDA)-approved drugs, was obtained from LTKB, a project at the FDA National Center for Toxicological Research (https://www.fda.gov/science-research/liver-toxicity-knowledge-base-ltkb/drug-induced-liver-injury-rank-dilirank-dataset). In the dataset, drugs are divided into four classes, ^v^Most-DILI-concern (192 drugs), ^v^Less-DILI-concern (278 drugs), ^v^No-DILI-concern (312 drugs), and Ambiguous-DILI-concern drugs (254 drugs), according to their potential for causing DILI. Among them, all the ^v^Most-DILI-concern, ^v^Less-DILI-concern and ^v^No-DILI-concern drugs were used as target compounds in this study, and ^v^Most-DILI-concern and ^v^Less-DILI-concern drugs were defined as DILI drugs (total of 470) and ^v^No-DILI-concern drugs were as no-DILI drugs.

The DILIrank dataset contains the lists of drug names and PubChem CIDs. Based on the CIDs, the CAS number of each drug was obtained, and their chemical structures were confirmed and defined using SciFinder (Chemical Abstracts Service, Columbus, OH) and PubChem (https://pubchem.ncbi.nlm.nih.gov). The salt compounds were desalted (e.g. hydrochloride of hydrochloride salt compounds was removed) or neutralized (e.g. sodium carboxylates were changed to carboxylic acids), and peptides, nucleic acids and metal-containing drugs were excluded in this study. The list of final target compounds (432 DILI drugs and 220 no-DILI drugs) is shown in S1 Table.

### Chemical descriptors

The SMILES format data of all the compounds used in this study were obtained from PubChem database. The SMILES were converted to MOL format using OpenBabelGUI [25]. The two-dimensional (2D) chemical descriptors were calculated with Dragon 7 software (Talete, Milano, Italy) with MOL format data. The descriptors that were incalculable and those that were constant among all the target compounds were excluded, and the remaining 2,473 descriptors (S2 Table) were used for analyses.

### Statistical analysis and machine learning

Statistical analyses and machine learning were performed using Microsoft Excel, JMP Pro 14 (SAS Institute, Cary, NC) and R (version 3.6.2 and 4.0.1) [26].

### Chemical space of target compounds

The chemical space of the target compounds (432 DILI drugs and 220 no-DILI drugs) was compared with that of 12,262 chemical compounds that were obtained from PubChem database as references, using principal component analysis (PCA). PubChem contains about 100 million small molecules with fewer than 1000 atoms and 1,000 bonds [27]. For comparison, 20,000 numbers (1–111,442,486) were randomly generated and these numbers were used as potential CIDs to search SMILES with PubChem Identifier Exchange Service (https://pubchem.ncbi.nlm.nih.gov/idexchange/idexchange.cgi). Finally, the valid SMILES format data of 12,262 chemical compounds were obtained. The chemical descriptors of these compounds (12,914 in total) were calculated. Those with zero or near-zero variance were excluded with the R function “nearZeroVar()” in the caret package, and further selection was performed considering linearity among descriptors using the R function “findLinearCombos()” in the caret package. The remaining 1,539 descriptors were subjected to PCA with the R function “prcomp()” in the stats package.

### Discriminant analysis using tree models

To build and validate decision tree models, nested cross-validation was performed as follows: The target compounds were randomly divided into 3 groups keeping the proportions of DILI drugs and no-DILI drugs same with the original proportion of the 652 drugs (432:220). Three trees were constructed using each pair of two groups as a training set and the rest was used as a test set. The decision trees that discriminate between DILI drugs and no-DILI drugs were constructed with the chemical descriptors as explanatory variables using the Classification and Regression Tree (CART) algorithm with the R function “rpart()” in the rpart package [22]. To determine the depth of each tree model, 5-fold cross-validation was carried out.

The final model was independently constructed with 5-fold cross-validation using the chemical descriptors of the 652 drugs. As the indices of prediction performance, accuracy, sensitivity, specificity, balanced accuracy (BA) and Matthews correlation coefficient (MCC), where TP, TN, FP, and FN represent the number of true positives, the number of true negatives, the number of false positives, and the number of false negatives, respectively:

$$\text{Accuracy}=\frac{\text{TP+TN}}{\text{total number of samples}},$$ (1)

$$\text{Sensitivity}=\frac{\text{TP}}{\text{TP+FN}},$$ (2)

$$\text{Specificity}=\frac{\text{TN}}{\text{TN+FP}},$$ (3)

$$\text{BA}=\frac{\text{sensitivity + specificity}}{\text{2}},$$ (4)

$$\text{MCC}=\frac{\text{TP}\times \text{TN}–\text{FP}\times \text{FN}}{\sqrt{\left(\text{TP+FP}\right)\times \left(\text{TP+FN}\right)\times \left(\text{TN+FP}\right)\times \left(\text{TN+FN}\right)}}.,$$ (5)

The receiver operating characteristic (ROC) curves were created using the ROCR package on R and the area under curve (AUC) was calculated based on the ROC curve.

For the final tree model, Mean Decrease in Gini [22] were calculated as importance scores for each variable. The score takes a value of 0 or more, and an explanatory variable with a large score is considered to make a substantial contribution to the construction of decision trees and discrimination of drugs.

### Categories of the Anatomical Therapeutic Chemical (ATC) classification system

Information on the ATC categories of the target compounds was obtained from KEGG drug database (https://www.genome.jp/kegg/drug/drug_ja.html).

### AMW of approved drugs

The information on drugs approved during the period from 2010 to 2019 in EU, USA and Japan was obtained from KEGG drug database. Peptides, nucleic acids and metal-containing drugs were excluded. Drugs marketed in multiple areas were labeled with the latest year.

## Results

### Selection of data set and its chemical space

**Fig 1:**
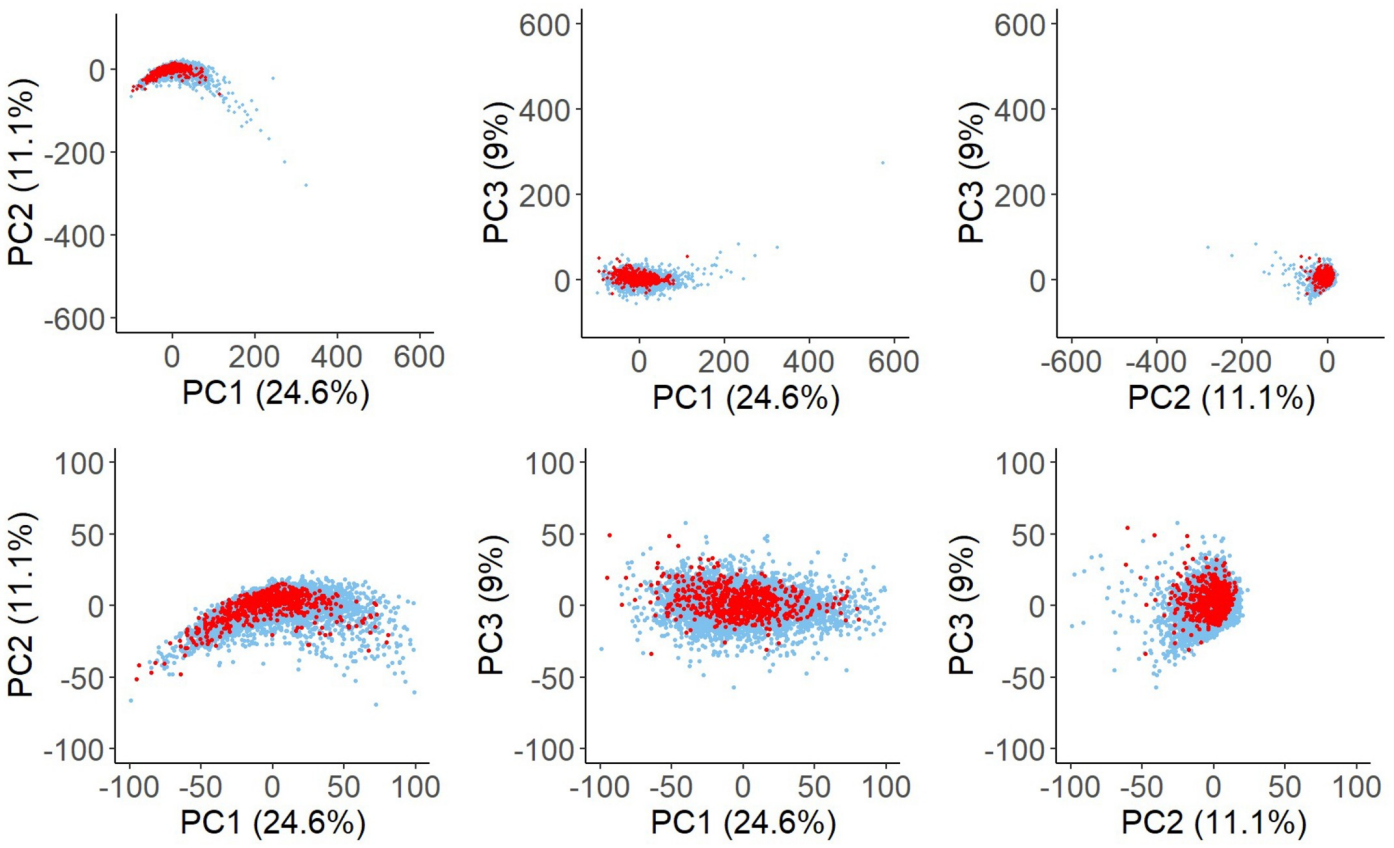
The PCA plots of the 652 target drugs and the 12,262 PubChem reference compounds. The PCA was performed using the 1,539 Dragon 7 descriptors. The plots of PC1 vs. PC2 (left), PC1 vs. PC3 (center) and PC2 vs. PC3 (right) are shown. The upper panels are the original views of all the compounds and the lower panels are the expanded views around the target drugs. Each dot represents a compound (red, target drugs; blue, PubChem compounds).

We obtained a total of 652 drugs, including 432 DILI drugs and 220 no-DILI drugs, as target compounds in this study. Using the Dragon 2D chemical descriptors calculated, the chemical space of these drugs was compared with that of 12,262 reference compounds, which were randomly selected from PubChem database, to understand whether the target compounds represent the chemical space of global chemical compounds. To this end, PCA was performed with the 1,539 descriptors of the 12,914 target and reference compounds, and scattered plots of all the combinations of principal component 1 (PC1), PC2 and PC3 are shown in Fig 1. The distribution of the target compounds well overlapped with that of the reference PubChem compounds. These results suggest that the 652 target drugs belong to the typical chemical space of known chemical compounds.

### Classification tree model for DILI drugs

**Fig 2:**
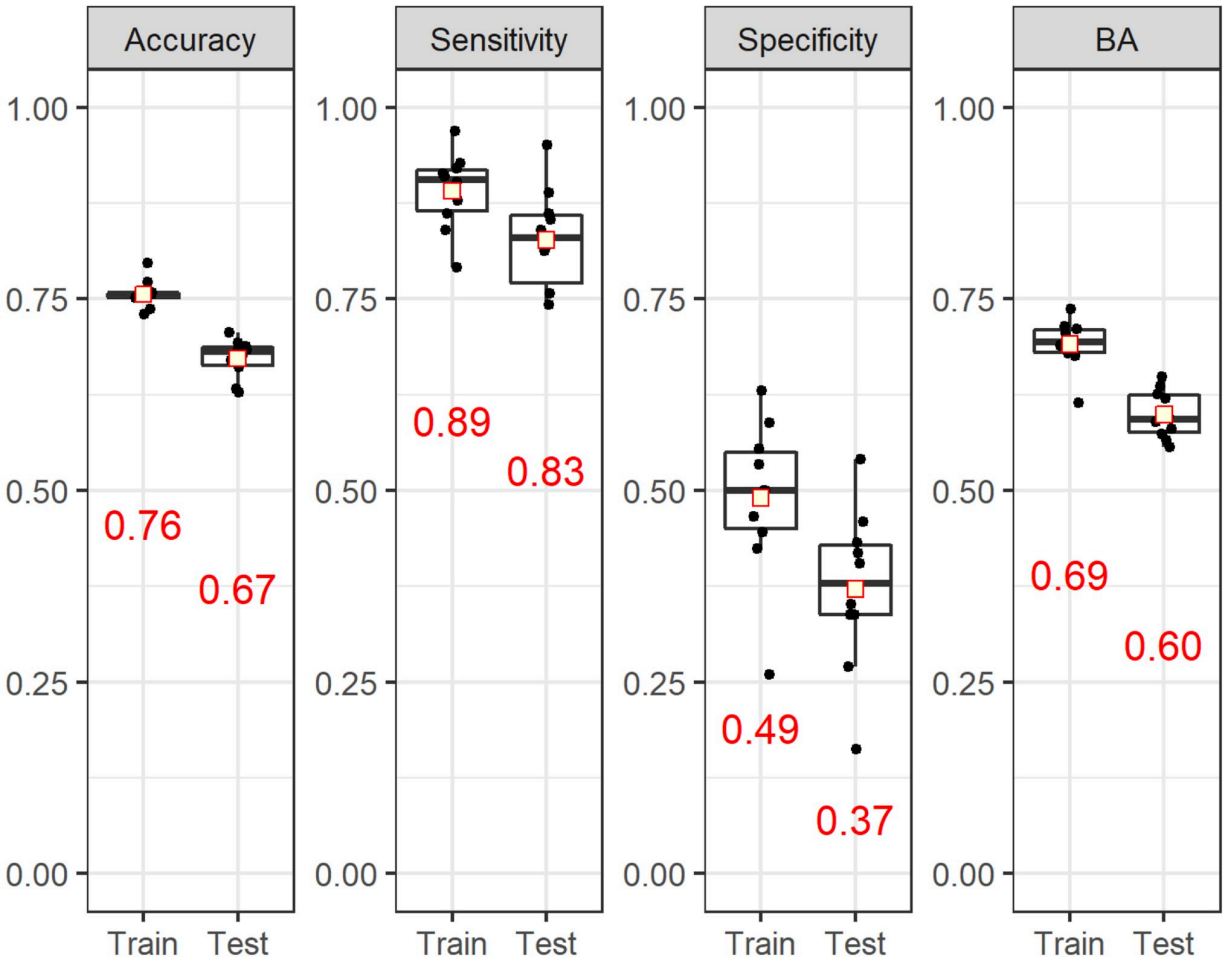
The performance of the classification models. The accuracy, sensitivity, specificity and BA were calculated from 10 trials of decision tree analysis with 5-fold cross-validation and are shown as box plots. The red numbers and squares represent the means of 10 trials with each value as black dots. Train, training set; Test, test set.

To investigate chemical properties associated with DILI, we constructed a classification model using the chemical descriptors as explanatory variables. The target compounds were divided into three groups, and using these subsets, three decision tree models were constructed with the CART algorithm with 5-fold cross-validation, and the accuracy, sensitivity, specificity and BA of the training data and test data were calculated. These steps were repeated 10 times and the means of the performance values are shown in Fig 2. The models showed high sensitivity (>0.8) and moderate accuracy (accuracy and BA, >0.6), but low specificity (<0.5).

**Fig 3:**
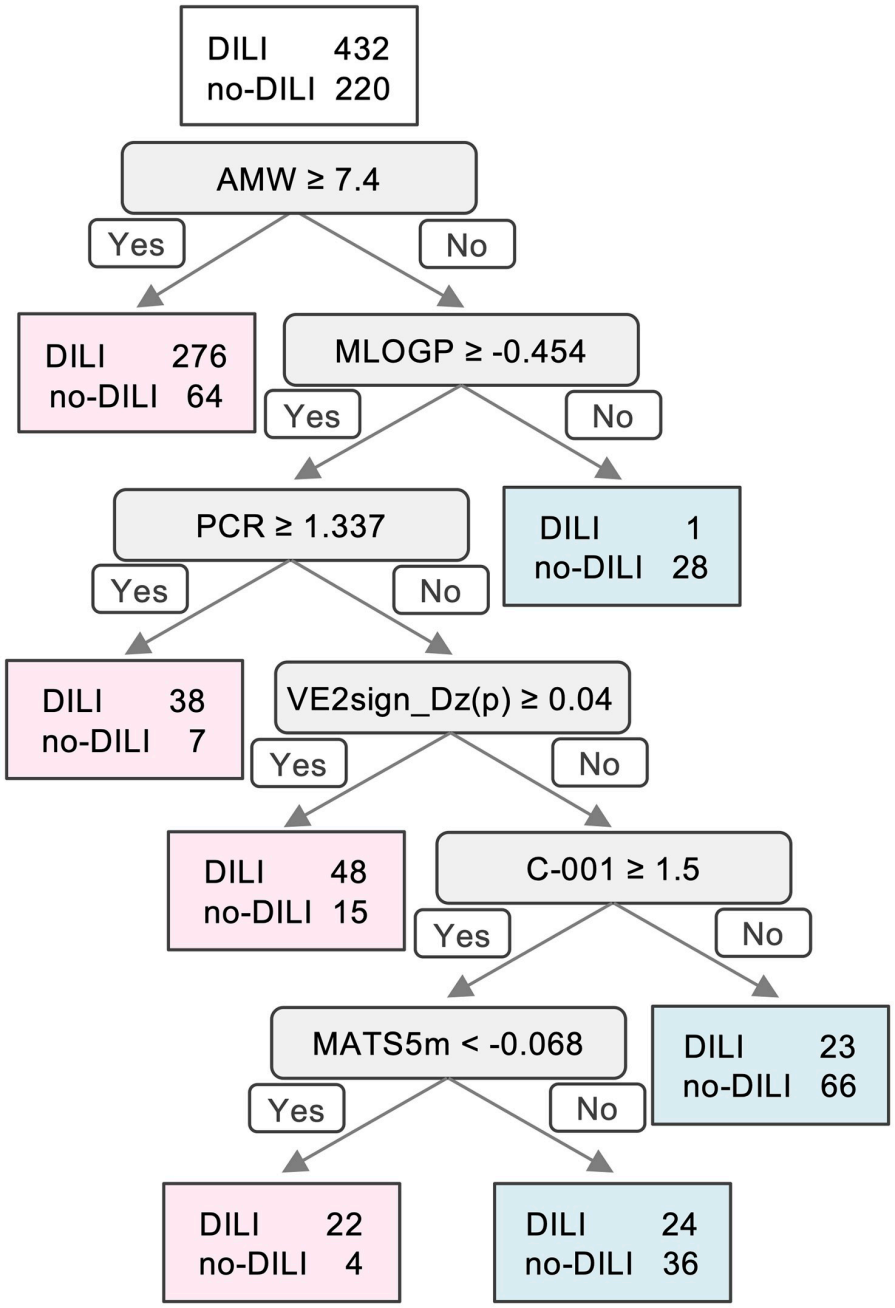
The final tree model for the discrimination between DILI drugs and no-DILI drugs. A decision tree model was constructed, and its accuracy, sensitivity, specificity and BA were calculated. Descriptor abbreviations are: PCR, ratio of multiple path count over path count; VE2sign_Dz(p), average coefficient of the last eigenvector from Barysz matrix weighted by polarizability; C-001, CH3R / CH4; MATS5m, Moran autocorrelation of lag 5 weighted by mass.

**Fig 4:**
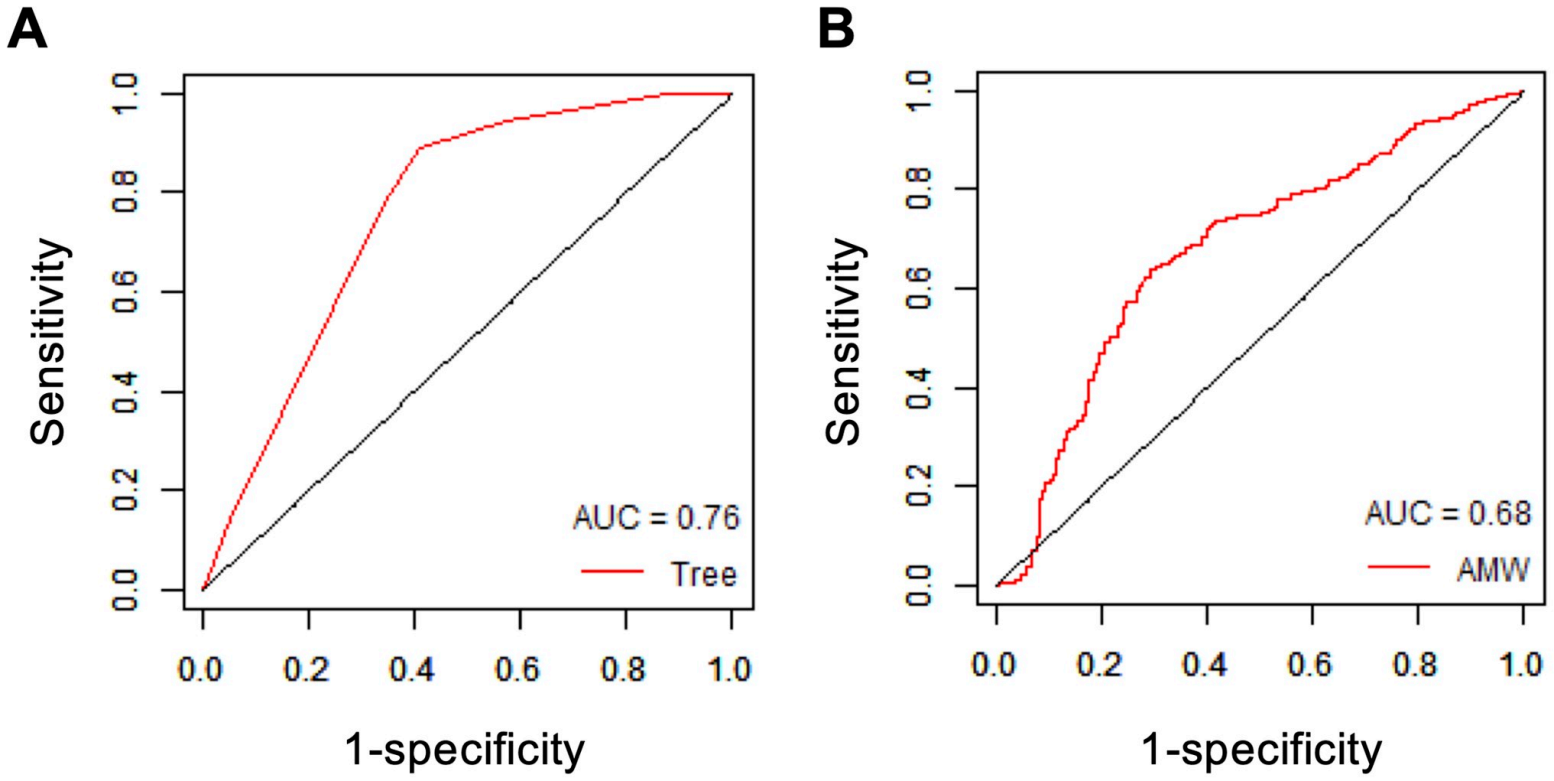
ROC analyses. ROC-AUCs were calculated by discriminating between DILI drugs and no-DILI drugs using the final tree model (A) and AMW alone as an index (B) as described in Materials and Methods.

The classification tree model independently constructed with 5-fold cross-validation using all the target compounds is shown in Fig 3. The confusion matrix that summarizes the results of testing the final tree model is shown as Table 1, and the importance values of the decision tree variables are shown in Table 2. The accuracy, sensitivity, specificity, BA, MCC and ROC-AUC of the model (Fig 4A) were 0.78, 0.89, 0.59, 0.74, 0.51 and 0.76, respectively. Although the specificity was relatively low as expected from the imbalanced dataset (i.e. DILI:no-DILI = 432:220), the model showed moderate discriminating performance.

**Table 1:** Confusion matrix of the final tree mode.

		Predicted	
		DILI	no-DILI
Actual	DILI	384	48
	no-DILI	90	130

**Table 2:** The importance values of the descriptors that were used for model construction.

Descriptor name	Description*	Block*	Importance
AMW	average molecular weight	Constitutional indices	31.628
GATS1m	Geary autocorrelation of lag 1 weighted by mass	2D autocorrelations	31.390
C-001	CH3R / CH4	Atom-centred fragments	30.108
SpPosA_B(m)	normalized spectral positive sum from Burden matrix weighted by mass	2D matrix-based descriptors	28.129
H%	percentage of H atoms	Constitutional indices	27.963
Mp	mean atomic polarizability (scaled on Carbon atom)	Constitutional indices	27.392
F09[O-O]	Frequency of O—O at topological distance 9	2D Atom Pairs	20.330
P_VSA_v_3	P_VSA-like on van der Waals volume, bin 3	P_VSA-like descriptor	19.919
MLOGP	Moriguchi octanol-water partition coeff. (logP)	Molecular properties	13.857
P_VSA_ppp_L	P_VSA-like on potential pharmacophore points, L–lipophilic	P_VSA-like descriptor	13.600
VE2sign_Dz(p)	average coefficient of the last eigenvector from Barysz matrix weighted by polarizability	2D matrix-based descriptors	12.521
BAC	Balaban centric index	Topological indices	12.151
SsssN	Sum of sssN E-states	Atom-type E-state indices	11.462
P_VSA_p_3	P_VSA-like on polarizability, bin 3	P_VSA-like descriptor	11.460
PCR	ratio of multiple path count over path count	Walk and path counts	9.423
PCD	difference between multiple path count and path count	Walk and path counts	8.866
SpDiam_AEA(ed)	spectral diameter from augmented edge adjacency mat. weighted by edge degree	Edge adjacency indices	8.515
MATS5m	Moran autocorrelation of lag 5 weighted by mass	2D autocorrelations	7.221
SsOH	Sum of sOH E-states	Atom-type E-state indices	6.921
NssCH2	Number of atoms of type ssCH2	Atom-type E-state indices	6.739
X1MulPer	multiplicative perturbation connectivity index	Connectivity indices	6.513
X1Per	perturbation connectivity index	Connectivity indices	6.513
VE1sign_B(i)	coefficient sum of the last eigenvector from Burden matrix weighted by ionization potential	2D matrix-based descriptors	6.429
CIC2	Complementary Information Content index (neighborhood symmetry of 2-order)	Information indices	6.238
MATS7i	Moran autocorrelation of lag 7 weighted by ionization potential	2D autocorrelations	6.151
nCp	number of terminal primary C(sp3)	Functional group counts	6.066

*The descriptions of the descriptors and the names of blocks (group of descriptors) were obtained from the Dragon 7 manual.

We found that the descriptor AMW, which represents average molecular weight, was the most important for discriminating DILI drugs from no-DILI drugs since it was used at the first node and showed the highest importance value (Fig 3, Table 2). The number of drugs whose AMW values are 7.4 or larger was 340, among which 276 (81%) were DILI drugs (Fig 3, Table 3).

**Table 3:** Contingency table between DILI/no-DILI and AMW.

		AMW		Total
		≥7.4	<7.4	
Most*	Count	120	55	175
	% within AMW	35.3%	17.6%	26.8%
Less*	Count	156	101	257
	% within AMW	45.9%	32.4%	39.4%
DILI	Count	276	156	432
	% within AMW	81.2%	50.0%	66.3%
no-DILI	Count	64	156	220
	% within AMW	18.8%	50.0%	33.7%
Total	Count	340	312	652
	% within AMW	100.0%	100.0%	100.0%

*Most and Less represent ^v^Most-DILI-concern and ^v^Less-DILI-concern drugs in DILIrank, respectively.

MLOGP, Moriguchi octanol-water partition coefficient [28, 29], was used at the second node and showed moderate importance (ranked 9th) (Fig 3, Table 2). The number of drugs whose AMW values are less than 7.4 and MLOGP values are less than -0.454 were 29, among which 28 (97%) were no-DILI drugs, indicating that MLOGP was important to detect no-DILI drugs in this model.

### Evaluation of AMW as a novel discriminator between DILI drugs and no-DILI drugs

We found AMW, which is calculated by dividing molecular weight by the number of atoms, as a useful discriminator for DILI drugs from no-DILI drugs based on the decision tree model. We thus performed ROC analyses with AMW alone and AUC of 0.68 was obtained (Fig 4B), suggesting that AMW is a good descriptor for the discrimination. In addition, AMW is easy to understand and calculate, and should be available in the early stage of drug development. We therefore evaluated its usefulness hereafter.

As shown in the contingency table (Table 3), the proportion of DILI drugs in the drugs having the AMW value of ≥7.4 was considerably high (81.2%) while that in the drugs having the AMW value of <7.4 was 50%. Moreover, among the 652 target drugs, 340 (52%; 276 DILI and 64 no-DILI drugs) have the AMW values of ≥7.4, and the proportion was increased to 64% (276 in 432) for DILI drugs only and that of no-DILI drugs only was decreased to 29% (64 in 220) (Table 3). In this study, we grouped drugs with ^v^Most-DILI-concern and ^v^Less-DILI-concern in DILIrank as DILI drugs and we found that the ratio of the numbers of drugs having the AMW value of ≥7.4 to those having the AMW value of <7.4 was higher for the drugs with ^v^Most-DILI-concern than those with ^v^Less-DILI-concern (Table 3). Taken together, AMW with a threshold of 7.4 enabled us to identify about two-thirds of DILI drugs with 71% specificity and 66% accuracy (276 plus 156 per 652 drugs).

Therapeutic category is generally available at the early stages of drug development. We thus investigated the applicability of the DILI/no-DILI discrimination using the combination of AMW and such information. To this end, the test drugs were sub-grouped by the first level category of the ATC classification system, which represents main anatomical/pharmacological groups (Table 4), and discrimination using “AMW ≥ 7.4” as a criterion was carried out. The number of drugs belonging to each ATC first level category and the calculated accuracy, sensitivity, specificity and BA are shown in Table 4. In categories B (blood and blood forming organs), J (antiinfectives for systemic use), M (musculo-skeletal system) and S (sensory organs), high precision discrimination was observed with all the four indices being over 0.7. On the other hand, in categories C (cardiovascular system), G (genito urinary system and sex hormones) and R (respiratory system), either sensitivity or specificity was below 50% and the accuracy was 60% or less. The lowest accuracy (accuracy, 35%; BA, 42%) was observed in category V (various).

**Table 4:** Accuracy of discrimination using AMW by ATC first level categories.

ATC first level	Accuracy	Sensitivity	Specificity	BA	Number of drugs	
					DILI	no-DILI
A. Alimentary tract and metabolism	75%	65%	89%	77%	49	39
B. Blood and blood forming organs	85%	89%	82%	85%	9	12
C. Cardiovascular system	54%	42%	79%	60%	62	31
D. Dermatologicals	72%	60%	100%	80%	26	11
G. Genito-urinary system and sex hormones	48%	32%	71%	52%	21	16
H. Systemic hormonal preparations, excluding sex hormones and insulins	55%	50%	60%	55%	6	5
J. Antiinfectives for systemic use	80%	80%	75%	78%	80	8
L. Antineoplastic and immunomodulating agents	68%	71%	50%	61%	53	10
M. Musculo-skeletal system	86%	90%	77%	83%	37	13
N. Nervous system	65%	52%	87%	69%	79	46
P. Antiparasitic products, insecticides and repellents	69%	69%	67%	68%	13	3
R. Respiratory system	60%	42%	70%	56%	14	29
S. Sensory organs	74%	75%	72%	74%	24	22
V. Various	35%	50%	33%	42%	2	19
Total	67%	63%	75%	69%	303	141

**Fig 5:**
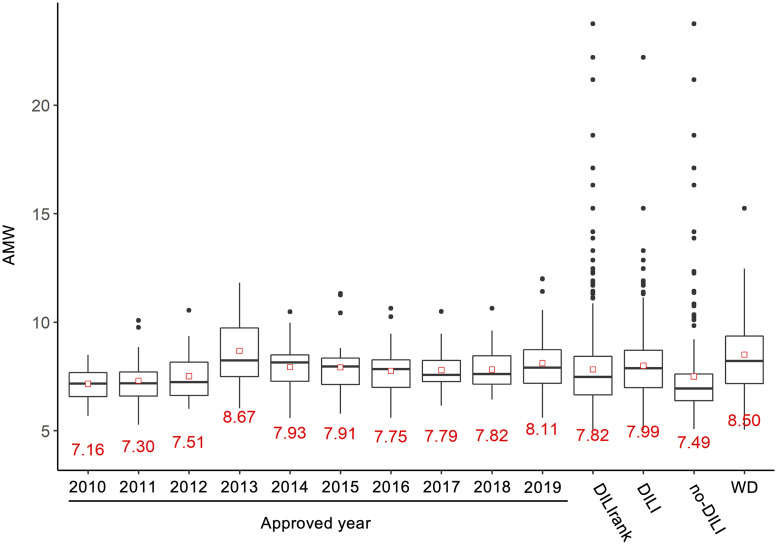
The trend of AMW of newly approved drugs. The AMW values of newly approved drugs for each year in EU, US and Japan were calculated. The numbers of drugs included are 18, 26, 29, 15, 38, 31, 30, 29, 40 and 50 in the year of 2010, 2011, 2012, 2013, 2014, 2015, 2016, 2017, 2018 and 2019, respectively. The list of drugs is shown in S3 Table. The values of the 652 drugs are also shown as references. The information of withdrawn drugs (WD) was obtained from DILIrank.

Finally, to evaluate the usefulness of AMW as a safety screening tool in drug development, we investigated the AMW values of newly approved drugs to understand the trend of the values (Fig 5). Among the drugs investigated (306 drugs), 179 (58%) have the AMW values of ≥7.4 (S3 Table), and the mean AMW values remained at around 7.9 during the last 10 years. With the exception of the year of 2013, the ranges of the AMW values were within that of the 652 target drugs when the outliers were excluded. It should be noted that the mean AMW value of the withdrawn drugs (53 drugs among the 432 DILI drugs) was much higher than the mean value of DILI drugs.

## Discussion

To identify chemical properties useful for discrimination between DILI drugs and no-DILI drugs, we constructed a decision tree model using the chemical descriptors as explanatory variables. The first node rule of the decision tree constructed was “DILI-positive if AMW is ≥7.4”, and we found that 64% of the DILI drugs were correctly classified by this criterion only and its AUC-ROC was 0.68. Moreover, only 29% of no-DILI drugs were found to have the AMW values of ≥7.4. In fact, the mean AMW values of DILI drugs and no-DILI drugs were 7.99 and 7.49, respectively, which were significantly different, and the mean AMW value of withdrawn drugs (8.50) was much higher than that of DILI drugs.

Among the descriptors used in this study, AMW (i.e. average molecular weight, which is calculated by dividing molecular weight by the number of atoms) is one of the simple descriptors, since it is easily calculated from the chemical formula of compounds without special software and knowledge on physicochemistry and computation. More importantly, AMW is available at the very early stage of drug development before chemical synthesis once target structures were determined. These facts suggest that AMW is a very simple and useful descriptor for discrimination between DILI drugs and no-DILI drugs and is applicable to medicinal chemistry to reduce hepatotoxicity potential during the drug development process.

The value of AMW becomes larger as a molecule contains more heavy atoms, such as halogens, oxygen, phosphorus and sulfur atoms. In contrast, a saturated molecule with a large number of hydrogen atoms has a smaller value. While many small-molecule drugs, such as those used in this study, are usually composed of hydrocarbon structures modified with nitrogen, oxygen, phosphorus, sulfur and halogen atoms, the presence of halogens, such as chlorine and iodine, especially contributes to a significant increase in AMW values. Since the inclusion of these atoms is often associated with toxic alert structures and/or reactive metabolite formation [30], large values of AMW may reflect the reactivity of drugs with biomolecule.

Therapeutic category information is generally available at the very early stages of drug development, and thus such information might be used as different types of descriptors from conventional descriptors, such as Dragon descriptors used in this study. With ATC classification as target disease information, the ATC first level categories, in combination with AMW, were used for discrimination. We found that the accuracy of discrimination by AMW was dependent on the categories. The accuracy was high for categories B, J, M and S while it was low for the others. Since drugs with similar chemical structures are often included in a certain ATC first level category, the results suggest that the discrimination accuracy with AMW is influenced by total and/or partial chemical structures or other factors of drugs. In addition, these results also suggest that the applicability of AMW for DILI discrimination is limited to drugs with specific but not yet identified chemical structures and/or pharmacological activity. Identification of these properties in future studies will corroborate and further increase the usefulness of AMW in the discrimination of DILI drugs.

A survey of the AMW values of newly approved drugs in EU, USA and Japan over the last 10 years (2010 to 2019) has shown that the average values have remained around 7.9 from 2013 to 2019, implying that a large number of the approved drugs have DILI risk (i.e. AMW ≥ 7.4) based on our present findings. Given that severe hepatotoxicity is reported for several drugs on the market, we believe that the criterion of “AMW ≥ 7.4” is also valuable to evaluate the hepatotoxicity potential of drugs on the market.

In the final decision tree model, the descriptor MLOGP was used at the second node. MLOGP is a calculated logP value by Moriguchi’s method and is reported to well explain experimentally determined logP values of more than a thousand compounds [28, 29]. The importance of the logP-related value for the discrimination of DILI drugs agrees with the previous findings that lipophilicity is a key characteristic of DILI drugs [13, 18].

As shown in Table 2, the importance values of AMW and GATS1m were very similar. Not only AMW but also GATS1m are considered to be descriptors related to DILI risk. In a very recent report using Dragon descriptors by Ancuceanu et al., GATS1m was frequently used in 17 feature selection algorithms based on the DILIrank dataset and its higher values are associated with the lack of hepatotoxicity [31]. In their study, SpPosA_B(m), H%, Mp, MLOGP and PCR were also reported as the descriptors associated with hepatotoxicity, some of which were used in our final decision tree model as well. However, AMW was not identified in their report. Although the exact reason is unknown, it may be due to a subtle difference in the set of drugs used in their and our studies.

There are several reports on in silico DILI prediction models using LTKB [31–34]. The accuracy of the models using a Decision Forest algorithm reported by Tong’s group at FDA were 69.7% and 72.9% [32, 33]. The ensemble voting model with various machine learning methods and several molecular fingerprints showed the accuracy of 77.25% (5-fold cross-validation) and 81.67% for the test set [33]. A Meta-model built with a variety of machine learning algorithms using Dragon descriptors showed similar accuracy (BA was 74.6%) [31]. In this study, the accuracy of the decision tree model with 5-fold cross-validation was 78%, and more importantly the discrimination with AMW only showed 66% accuracy, indicating AMW as a very useful single predictor for DILI drugs, although there are some limitations of the utility of AMW. First, AMW was identified from the limited number of drugs (652 drugs in the DILIrank dataset of LTKB) and we have not performed validation with an external dataset because no such a dataset is publicly available. Second, the discrimination accuracy (66%), sensitivity (64%) and specificity (71%) are not high enough to use AMW alone for drug screening and it may need to be used in combination with other descriptors. Finally, the applicability domain of AMW has not been identified. Based on the results obtained by the analyses using the ATC category, AMW is suggested to be applicable to only some types of drugs since the accuracy of the classification with AMW largely depended on the category.

In conclusion, using decision tree analyses with chemical descriptors, we obtained useful information of physicochemical properties of drugs for DILI discrimination. In particular, AMW has been found to be a useful chemical descriptor for DILI discrimination. Since calculation of AMW does not require chemical synthesis of candidate compounds, the consideration of AMW will be very valuable for DILI risk evaluation at the very early stage of drug discovery. Moreover, we found that the utilization of the disease area information (i.e. ATC classification information) increased the accuracy of DILI prediction using AMW. Taken together, the present findings provide useful information that is applicable to the safety assessment of drug candidates at the early stage of drug development.

## Supporting information

S1 Table The list of target compounds used. (XLSX)

S2 Table The list of chemical descriptors used. (XLSX)

S3 Table The list of newly approved drugs used and their AMW values. (XLSX)

## References

[1] US Food and Drug Administration. Guidance for Industry. Drug-Induced Liver Injury: Premarketing Clinical Evaluation. 2009.

[2] Wilke RA, Lin DW, Roden DM, Watkins PB, Flockhart D, Zineh I, et al. Identifying genetic risk factors for serious adverse drug reactions: Current progress and challenges. Nat Rev Drug Discov. 2007; 6: 904–916. doi: 10.1038/nrd2423 17971785 PMC2763923

[3] Cook D, Brown D, Alexander R, March R, Morgan P, Satterthwaite G, et al. Lessons learned from the fate of AstraZeneca’s drug pipeline: A five-dimensional framework. Nat Rev Drug Discov. 2014; 13: 419–431. doi: 10.1038/nrd4309 24833294

[4] Garcia-Cortes M, Robles-Diaz M, Stephens C, Ortega-Alonso A, Lucena MI, Andrade RJ. Drug induced liver injury: an update. Arch Toxicol. 2020; 94: 3381–3407. doi: 10.1007/s00204-020-02885-1 32852569

[5] Kaplowitz N. Idiosyncratic drug hepatotoxicity. Nat Rev Drug Discov. 2005; 4: 489–499. doi: 10.1038/nrd1750 15931258

[6] Park BK, Boobis A, Clarke S, Goldring CEP, Jones D, Kenna JG, et al. Managing the challenge of chemically reactive metabolites in drug development. Nat Rev Drug Discov. 2011; 10: 292–306. doi: 10.1038/nrd3408 21455238

[7] Chalasani N, Björnsson E. Risk factors for idiosyncratic drug-induced liver injury. Gastroenterology 2010; 138: 2246–2259. doi: 10.1053/j.gastro.2010.04.001 20394749 PMC3157241

[8] Li X, Gao P, Niu J. Metabolic comorbidities and risk of development and aeverity of drug-induced liver injury. Biomed Res Int. 2019; 2019: 1–9. doi: 10.1155/2019/8764093 31531370 PMC6720367

[9] Kato R, Uetrecht J. Supernatant from hepatocyte cultures with drugs that cause idiosyncratic liver injury activates macrophage inflammasomes. Chem Res Toxicol. 2017; 30: 1327–1332. doi: 10.1021/acs.chemrestox.7b00065 28525267

[10] Hirata K, Takagi H, Yamamoto M, Matsumoto T, Nishiya T, Mori K, et al. Ticlopidine-induced hepatotoxicity is associated with specific human leukocyte antigen genomic subtypes in Japanese patients: a preliminary case–control study. Pharmacogenomics J. 2008; 8: 29–33. doi: 10.1038/sj.tpj.6500442 17339877

[11] Garside H, Marcoe KF, Chesnut-Speelman J, Foster AJ, Muthas D, Kenna JG, et al. Evaluation of the use of imaging parameters for the detection of compound-induced hepatotoxicity in 384-well cultures of HepG2 cells and cryopreserved primary human hepatocytes. Toxicol In Vitro. 2014; 28: 171–181. doi: 10.1016/j.tiv.2013.10.015 24189122

[12] Endo S, Toyoda Y, Fukami T, Nakajima M, Yokoi T. Stimulation of human monocytic THP-1 cells by metabolic activation of hepatotoxic drugs. Drug Metab Pharmacokinet. 2012; 27: 621–630. doi: 10.2133/dmpk.dmpk-12-rg-019 22785256

[13] Chen M, Borlak J, Tong W. High lipophilicity and high daily dose of oral medications are associated with significant risk for drug-induced liver injury. Hepatology 2013; 58: 388–396. doi: 10.1002/hep.26208 23258593

[14] Lammert C, Einarsson S, Saha C, Niklasson A, Bjornsson E, Chalasani N. Relationship between daily dose of oral medications and idiosyncratic drug-induced liver injury: Search for signals. Hepatology 2008; 47: 2003–2009. doi: 10.1002/hep.22272 18454504

[15] Lammert C, Bjornsson E, Niklasson A, Chalasani N. Oral medications with significant hepatic metabolism at higher risk for hepatic adverse events. Hepatology 2010; 51: 615–620. doi: 10.1002/hep.23317 19839004

[16] Chen M, Suzuki A, Borlak J, Andrade RJ, Lucena MI. Drug-induced liver injury: Interactions between drug properties and host factors. J Hepatol. 2015; 63: 503–514. doi: 10.1016/j.jhep.2015.04.016 25912521

[17] McEuen K, Borlak J, Tong W, Chen M. Associations of drug lipophilicity and extent of metabolism with drug-induced liver injury. Int J Mol Sci. 2017; 18: 1335. doi: 10.3390/ijms18071335 28640208 PMC5535828

[18] Chen M, Tung CW, Shi Q, Guo L, Shi L, Fang H, et al. A testing strategy to predict risk for drug-induced liver injury in humans using high-content screen assays and the ‘rule-of-two’ model. Arch Toxicol. 2014; 88: 1439–1449. doi: 10.1007/s00204-014-1276-9 24958025 PMC5753582

[19] Hosoya R, Uesawa Y, Ishii-Nozawa R, Kagaya H. Analysis of factors associated with hiccups based on the Japanese Adverse Drug Event Report database. PLoS One. 2017; 12: e0172057. doi: 10.1371/journal.pone.0172057 28196104 PMC5308855

[20] Liu J, Mansouri K, Judson RS, Martin MT, Hong H, Chen M, et al. Predicting hepatotoxicity using ToxCast in vitro bioactivity and chemical Structure. Chem Res Toxicol. 2015; 28: 738–751. doi: 10.1021/tx500501h 25697799

[21] Low Y, Uehara T, Minowa Y, Yamada H, Ohno Y, Urushidani T, et al. predicting drug-induced hepatotoxicity using QSAR and toxicogenomics approaches. Chem Res Toxicol. 2011; 24: 1251–1262. doi: 10.1021/tx200148a 21699217 PMC4281093

[22] Therneau TM, Atkinson EJ. An Introduction to recursive partitioning using the RPART routines. Mayo Clin Sect Biostat Tech Rep. 2019; 61: 33.

[23] Chen M, Vijay V, Shi Q, Liu Z, Fang H, Tong W. FDA-approved drug labeling for the study of drug-induced liver injury. Drug Discov Today. 2011; 16: 697–703. doi: 10.1016/j.drudis.2011.05.007 21624500

[24] Chen M, Suzuki A, Thakkar S, Yu K, Hu C, Tong W. DILIrank: The largest reference drug list ranked by the risk for developing drug-induced liver injury in humans. Drug Discov Today. 2016; 21: 648–653. doi: 10.1016/j.drudis.2016.02.015 26948801

[25] O’Boyle NM, Banck M, James CA, Morley C, Vandermeersch T, Hutchison GR. Open Babel: An open chemical toolbox. J Cheminform. 2011; 3: 33. doi: 10.1186/1758-2946-3-33 21982300 PMC3198950

[26] R Core Team. R: A language and environment for statistical computing. R Foundation for Statistical Computing, Vienna, Austria. (https://www.R-project.org/). 2020.

[27] Kim S, Thiessen PA, Bolton EE, Chen J, Fu G, Gindulyte A, et al. PubChem substance and compound databases. Nucleic Acids Res. 2016; 44: D1202–D1213. doi: 10.1093/nar/gkv951 26400175 PMC4702940

[28] Moriguchi I, Hirono S, Liu Q, Nakagome I, Matsushita Y. Simple method of calculating octanol/water partition coefficient. Chem Pharm Bull. 1992; 40: 127–130.

[29] Moriguchi I, Hirono S, Nakagome I, Hirano H. Comparison of reliability of log P values for drugs calculated by several methods. Chem Pharm Bull. 1994; 42: 976–978.

[30] Stepan AF, Walker DP, Bauman J, Price DA, Baillie TA, Kalgutkar AS, et al. Structural alert/reactive metabolite concept as applied in medicinal chemistry to mitigate the risk of idiosyncratic drug toxicity: A perspective based on the critical examination of trends in the top 200 drugs marketed in the United States. Chem Res Toxicol. 2011; 24: 1345–1410. doi: 10.1021/tx200168d 21702456

[31] Ancuceanu R, Hovanet MV, Anghel AI, Furtunescu F, Neagu M, Constantin C, et al. Computational models using multiple machine learning algorithms for predicting drug hepatotoxicity with the DILIrank dataset. Int J Mol Sci. 2020; 21: 2114. doi: 10.3390/ijms21062114 32204453 PMC7139829

[32] Chen M, Hong H, Fang H, Kelly R, Zhou G, Borlak J, et al. Quantitative structure-activity relationship models for predicting drug-induced liver injury based on FDA-approved drug labeling annotation and using a large collection of drugs. Toxicol Sci. 2013; 136: 242–249. doi: 10.1093/toxsci/kft189 23997115

[33] Hong H, Thakkar S, Chen M, Tong W. Development of Decision Forest models for prediction of drug-induced liver injury in humans using a large set of FDA-approved drugs. Sci Rep. 2017; 7: 17311. doi: 10.1038/s41598-017-17701-7 29229971 PMC5725422

[34] Wang Y, Xiao Q, Chen P, Wang B. In silico prediction of drug-induced liver injury based on ensemble classifier method. Int J Mol Sci. 2019; 20: 4106. doi: 10.3390/ijms20174106 31443562 PMC6747689

